# Supplementary material for: Kidney Bean Fermented Broth Alleviates Hyperlipidemic by Regulating Serum Metabolites and Gut Microbiota Composition
Source: Nutrients. 2022 Aug 5;14(15):3202. doi: 10.3390/nu14153202 (PMC9370468; doi:10.3390/nu14153202)
Supplement: Supplementary file 1 [file nutrients-14-03202-s001.zip › nutrients-1810407-SI.pdf]

Table S1 Effect of the KBF on body weight gain, feed intake and food efficiency ratio

		0 week	2 weeks	4 weeks	6 weeks	8 weeks
Body weight (g)	CON	254.98 ± 7.77	279.66 ± 7.58	307.43 ± 9.35	337.17 ± 11.94	373.28 ± 8.73
	MOD	254.17 ± 7.03	302.53 ± 18.73 **	352.92 ± 18.63 *	402.98 ± 6.54 **	452.31 ± 13.73 **
	KBF-L	257.90 ± 6.32	296.18 ± 7.64 **▲	343.42 ± 6.12 *▲▲	397.85 ± 6.84 **	441.10 ± 13.65 **▲▲
	KBF-M	256.15 ± 7.18	289.45 ± 10.29 *▲▲	338.25 ± 15.07 *▲▲	392.07 ± 10.27 **▲	437.73 ± 16.43 **▲▲
	KBF-H	256.68 ± 7.72	293.05 ± 9.67 **▲▲	329.67 ± 11.55 *▲▲	381.63 ± 5.48 **▲	428.57 ± 7.08 **▲▲
Body weight gain (g)	CON		24.68 ± 3.23	27.77 ± 2.38	29.74 ± 2.79	36.11 ± 1.13
	MOD		48.36 ± 2.15 **	50.39 ± 3.79 **	50.06 ± 2.06 **	49.33 ± 2.21 **
	KBF-L		38.28 ± 3.49 **▲▲	47.24 ± 2.08 ** ▲	54.43 ± 3.36 ** ▲	43.25 ± 1.32 **▲
	KBF-M		33.30 ± 2.54 **▲▲	48. 80 ± 2.22 **	53.82 ± 1.55 **▲	45.66 ± 1.64 **▲
	KBF-H		36.37 ± 2.60 **▲▲	36.62 ± 3.27 **▲▲	51.96 ± 0.46 **	46.94 ± 1.20**▲
Feed intake(g/d)	CON		159.87 ± 1.44	158.94 ± 4.47	160.00 ± 1.00	157.89 ± 1.06
	MOD		157.90 ± 1.55	160.31 ± 2.16	158.31 ± 4.16	158.12 ± 7.85
	KBF-L		160.10 ± 3.45	157.69 ± 8.81	156.91 ± 3.70	153.22 ± 6.54
	KBF-M		158.11 ± 1.81	158.63±3.42	157.44±1.52	155.23±7.14
	KBF-H		159.25 ± 0.13	157.00 ± 6.05	156.29 ± 6.74	155.37 ± 2.32
Food efficiency ratio	CON		0.15	0.17	0.19	0.23
	MOD		0.31	0.31	0.32	0.31
	KBF-L		0.24	0.30	0.35	0.28
	KBF-M		0.21	0.31	0.34	0.29
	KBF-H		0.23	0.23	0.33	0.30

Values are presented as the mean ± SD (n = 6). Significant difference compared with the MOD group. *p < 0.05, **p < 0.01. Significant difference compared with the CON group. ▲p < 0.05, ▲▲p < 0.01.

Table S2 Effect of the KBF on visceral organ quality, body length and abdominal circumference of rats

Group	body length/cm	abdominal circumference/cm	heart/g	liver /g	pancreas /g	kidney/g	spleen/g	epididymal visceral adipose (g)	brown fat (g)
CON	22.30 ± 1.15	15.93 ± 1.09	1.36 ± 0.01	9.97±0.77	1.18 ± 0.09	2.72 ± 0.12	0.75 ± 0.10	3.33 ± 0.15	0.54 ± 0.03
MOD	24.37 ± 0.90	18.27 ± 0.88	1.83 ± 0.12 ▲	14.08 ± 0.45 ▲▲	1.60 ± 0.02 ▲▲	3.68 ± 0.09 ▲▲	1.11 ± 0.12 ▲▲	6.56 ± 1.28 ▲▲	0.88 ± 0.03 ▲▲
KBF-L	24.20 ± 0.29	17.73 ± 0.37	1.52 ± 0.23	13.67 ± 1.58	1.53 ± 0.03	3.69 ± 0.12	0.92 ± 0.05	3.58 ± 0.30 **	0.72 ± 0.04 **
KBF-M	23.23 ± 0.45	17.13 ± 1.07	1.48 ± 0.07	12.03 ± 0.75	1.46 ± 0.02	3.35 ± 0.23	0.75 ± 0.05 **	2.65 ± 0.16 **	0.63 ± 0.03 **
KBF-H	22.80 ± 0.29	16.50±0.73	1.40 ± 0.03 *	10.36 ± 0.29 *	1.38 ± 0.02 *	3.03 ± 0.03 **	0.72±0.06**	2.19 ± 0.12 **	0.41 ± 0.04 **

Values are presented as the mean ± SD (n = 6). Significant difference compared with the CON group. ▲$p < 0.05$, ▲▲$p < 0.01$. Significant difference compared with the MOD group. *$p < 0.05$, **$p < 0.01$.

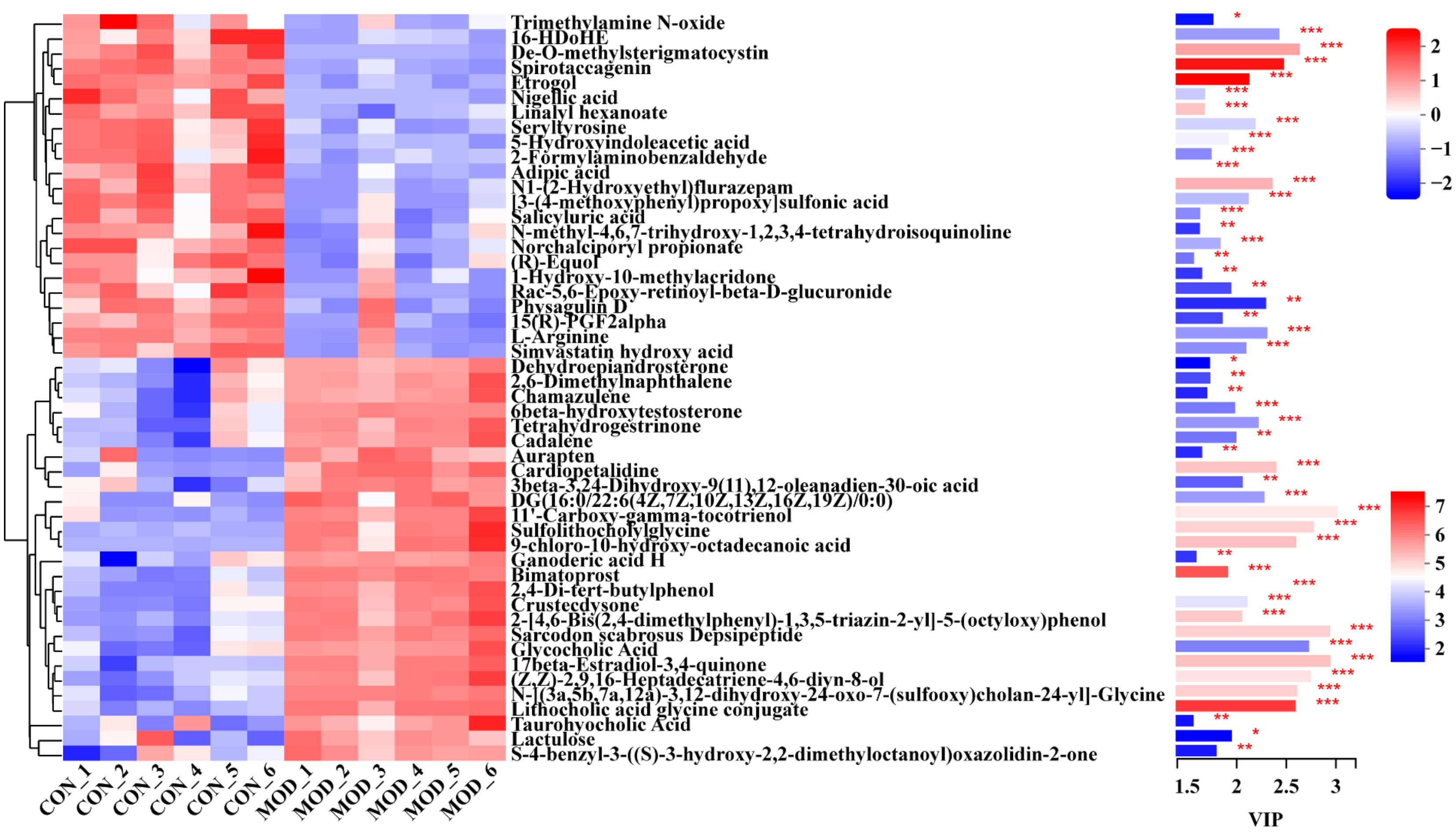
Trimethylamine N-oxide
16-HDoHE
De-O-methylsterigmatocystin
Spirotaccagenin
Etrogol
Nigellic acid
Linalyl hexanoate
Seryltyrosine
5-Hydroxyindoleacetic acid
2-Formylaminobenzaldehyde
Adipic acid
N1-(2-Hydroxyethyl)flurazepam
[3-(4-methoxyphenyl)propoxy]sulfonic acid
Salicyluric acid
N-methyl-4,6,7-trihydroxy-1,2,3,4-tetrahydroisoquinoline
Norchalciporyl propionate
(R)-Equol
1-Hydroxy-10-methylacridone
Rac-5,6-Epoxy-retinoyl-beta-D-glucuronide
Physagulin D
15(R)-PGF2alpha
L-Arginine
Simvastatin hydroxy acid
Dehydroepiandrosterone
2,6-Dimethylnaphthalene
Chamazulene
6beta-hydroxytestosterone
Tetrahydrogestrinone
Cadalene
Aurapten
Cardiopetalidine
3beta-3,24-Dihydroxy-9(11),12-oleanadien-30-oic acid
DG(16:0/22:6(4Z,7Z,10Z,13Z,16Z,19Z)/0:0)
11'-Carboxy-gamma-tocotrienol
Sulfolithocholylglycine
9-chloro-10-hydroxy-octadecanoic acid
Ganoderic acid H
Bimatoprost
2,4-Di-tert-butylphenol
Crustecdysone
2-[4,6-Bis(2,4-dimethylphenyl)-1,3,5-triazin-2-yl]-5-(octyloxy)phenol
Sarcodon scabrosus Depsipeptide
Glycocholic Acid
17beta-Estradiol-3,4-quinone
(Z,Z)-2,9,16-Heptadecatriene-4,6-diyn-8-ol
N-[(3a,5b,7a,12a)-3,12-dihydroxy-24-oxo-7-(sulfooxy)cholan-24-yl]-Glycine
Lithocholic acid glycine conjugate
Taurohyocholic Acid
Lactulose
S-4-benzyl-3-((S)-3-hydroxy-2,2-dimethyloctanoyl)oxazolidin-2-one
CON_1
CON_2
CON_3
CON_4
CON_5
CON_6
MOD_1
MOD_2
MOD_3
MOD_4
MOD_5
MOD_6
1.5
2
2.5
3
VIP
2
1
0
−1
−2
7
6
5
4
3
2

（a）

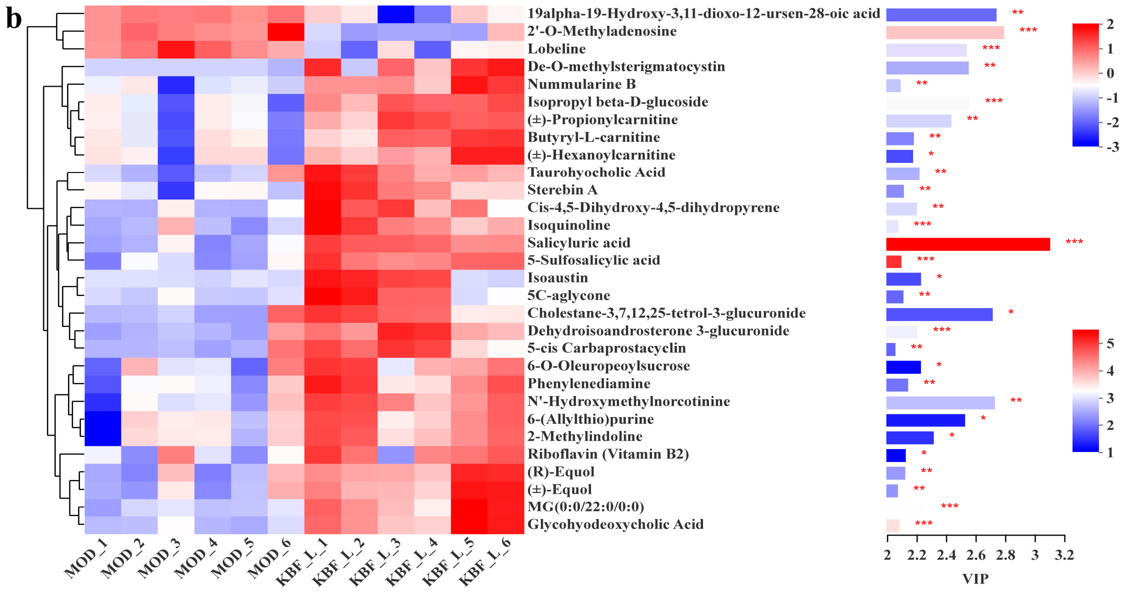
b
19alpha-19-Hydroxy-3,11-dioxo-12-ursen-28-oic acid
2'-O-Methyladenosine
Lobeline
De-O-methylsterigmatocystin
Nummularine B
Isopropyl beta-D-glucoside
(±)-Propionylcarnitine
Butyryl-L-carnitine
(±)-Hexanoylcarnitine
Taurohyocholic Acid
Sterebin A
Cis-4,5-Dihydroxy-4,5-dihydropyrene
Isoquinoline
Salicyluric acid
5-Sulfosalicylic acid
Isoaustin
5C-aglycone
Cholestane-3,7,12,25-tetrol-3-glucuronide
Dehydroisoandrosterone 3-glucuronide
5-cis Carbaprostacyclin
6-O-Oleuropeoylsucrose
Phenylenediamine
N'-Hydroxymethylnorcotinine
6-(Allylthio)purine
2-Methylindoline
Riboflavin (Vitamin B2)
(R)-Equol
(±)-Equol
MG(0:0/22:0/0:0)
Glycohyodeoxycholic Acid
MOD_1
MOD_2
MOD_3
MOD_4
MOD_5
MOD_6
KBF_L_1
KBF_L_2
KBF_L_3
KBF_L_4
KBF_L_5
KBF_L_6
VIP
2
2.2
2.4
2.6
2.8
3
3.2
2
1
0
-1
-2
-3
5
4
3
2
1

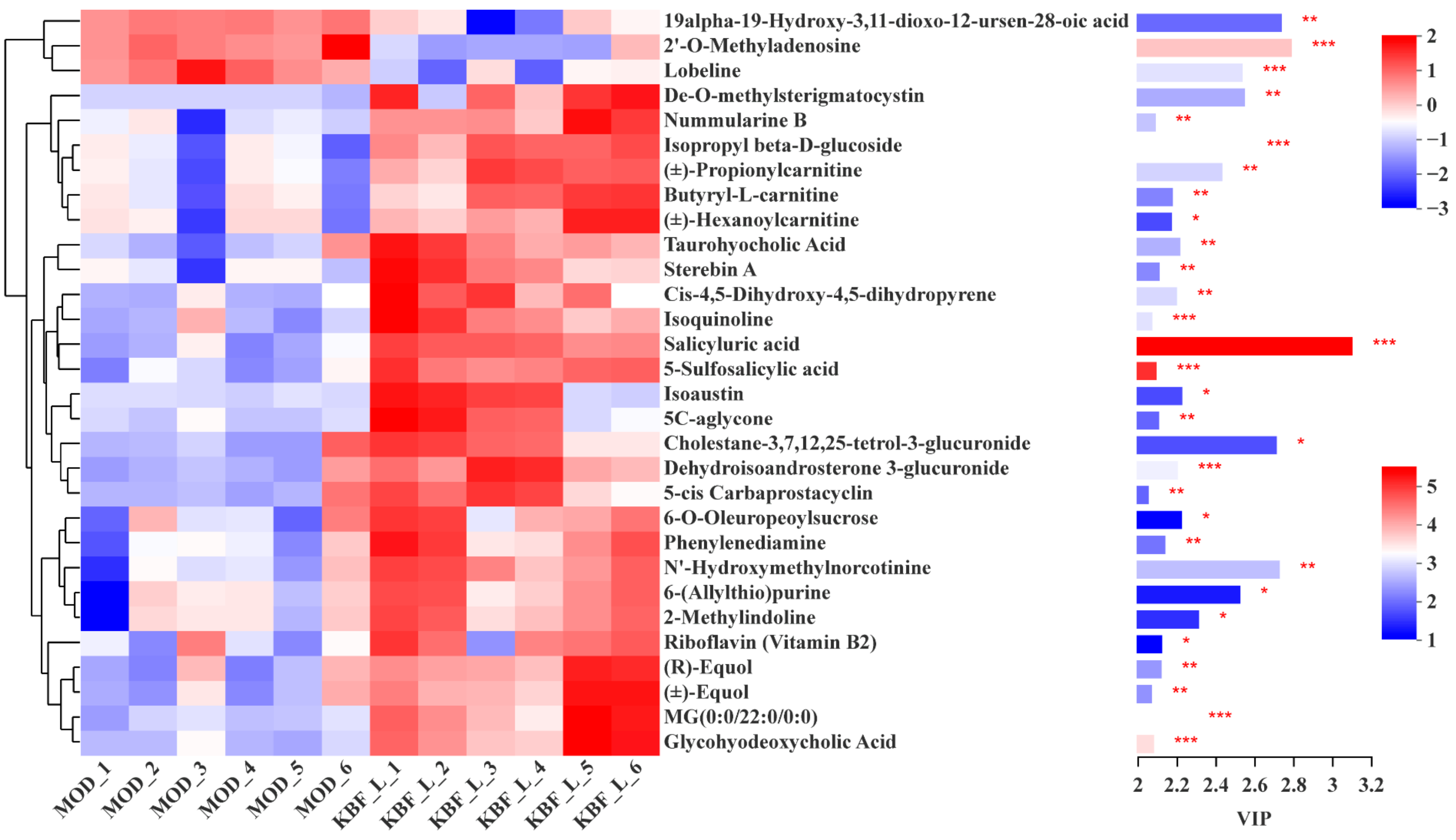
19alpha-19-Hydroxy-3,11-dioxo-12-ursen-28-oic acid
2'-O-Methyladenosine
Lobeline
De-O-methylsterigmatocystin
Nummularine B
Isopropyl beta-D-glucoside
(±)-Propionylcarnitine
Butyryl-L-carnitine
(±)-Hexanoylcarnitine
Taurohyocholic Acid
Sterebin A
Cis-4,5-Dihydroxy-4,5-dihydropyrene
Isoquinoline
Salicyluric acid
5-Sulfosalicylic acid
Isoaustin
5C-aglycone
Cholestane-3,7,12,25-tetrol-3-glucuronide
Dehydroisoandrosterone 3-glucuronide
5-cis Carbaprostacyclin
6-O-Oleuropeoylsucrose
Phenylenediamine
N'-Hydroxymethylnorcotinine
6-(Allylthio)purine
2-Methylindoline
Riboflavin (Vitamin B2)
(R)-Equol
(±)-Equol
MG(0:0/22:0/0:0)
Glycohyodeoxycholic Acid
MOD_1 MOD_2 MOD_3 MOD_4 MOD_5 MOD_6 KBF_L_1 KBF_L_2 KBF_L_3 KBF_L_4 KBF_L_5 KBF_L_6
VIP
2 2.2 2.4 2.6 2.8 3 3.2
2 1 0 −1 −2 −3
5 4 3 2 1
（b）

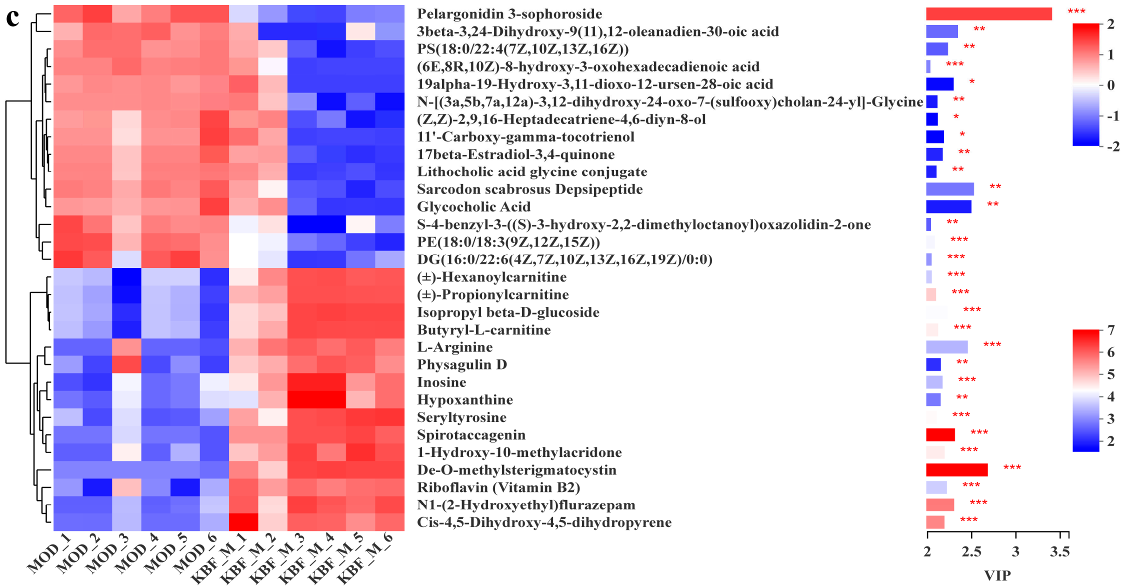
c
Pelargonidin 3-sophoroside
3beta-3,24-Dihydroxy-9(11),12-oleanadien-30-oic acid
PS(18:0/22:4(7Z,10Z,13Z,16Z))
(6E,8R,10Z)-8-hydroxy-3-oxohexadecadienoic acid
19alpha-19-Hydroxy-3,11-dioxo-12-ursen-28-oic acid
N-[(3a,5b,7a,12a)-3,12-dihydroxy-24-oxo-7-(sulfooxy)cholan-24-yl]-Glycine
(Z,Z)-2,9,16-Heptadecatriene-4,6-diyn-8-ol
11'-Carboxy-gamma-tocotrienol
17beta-Estradiol-3,4-quinone
Lithocholic acid glycine conjugate
Sarcodon scabrosus Depsipeptide
Glycocholic Acid
S-4-benzyl-3-((S)-3-hydroxy-2,2-dimethyloctanoyl)oxazolidin-2-one
PE(18:0/18:3(9Z,12Z,15Z))
DG(16:0/22:6(4Z,7Z,10Z,13Z,16Z,19Z)/0:0)
(±)-Hexanoylcarnitine
(±)-Propionylcarnitine
Isopropyl beta-D-glucoside
Butyryl-L-carnitine
L-Arginine
Physagulin D
Inosine
Hypoxanthine
Seryltyrosine
Spirotaccagenin
1-Hydroxy-10-methylacridone
De-O-methylsterigmatocystin
Riboflavin (Vitamin B2)
N1-(2-Hydroxyethyl)flurazepam
Cis-4,5-Dihydroxy-4,5-dihydropyrene
MOD_1
MOD_2
MOD_3
MOD_4
MOD_5
MOD_6
KBF_M_1
KBF_M_2
KBF_M_3
KBF_M_4
KBF_M_5
KBF_M_6
2
1
0
-1
-2
7
6
5
4
3
2
2
2.5
3
3.5
VIP

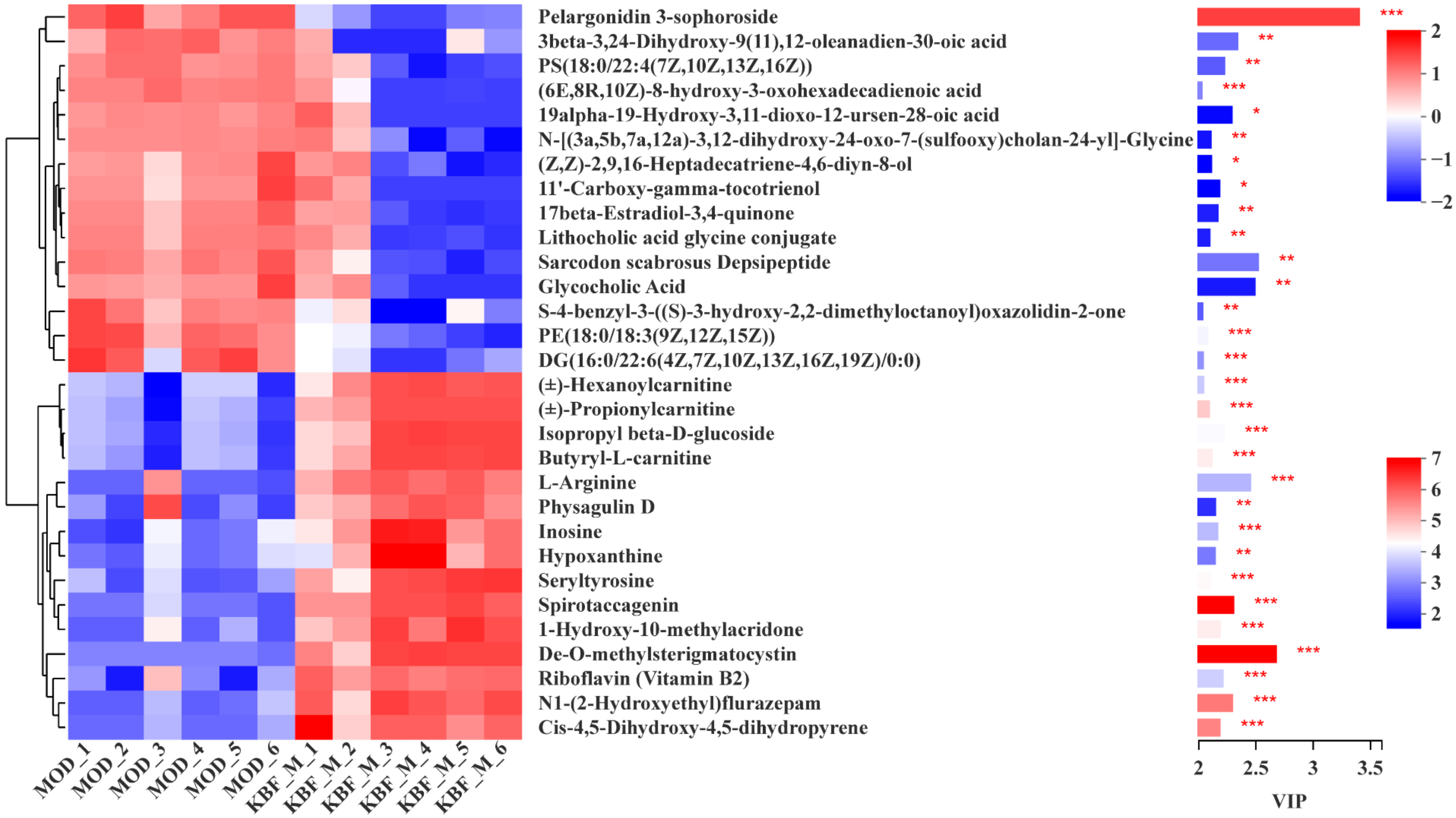
Pelargonidin 3-sophoroside
3beta-3,24-Dihydroxy-9(11),12-oleanadien-30-oic acid
PS(18:0/22:4(7Z,10Z,13Z,16Z))
(6E,8R,10Z)-8-hydroxy-3-oxohexadecadienoic acid
19alpha-19-Hydroxy-3,11-dioxo-12-ursen-28-oic acid
N-[(3a,5b,7a,12a)-3,12-dihydroxy-24-oxo-7-(sulfooxy)cholan-24-yl]-Glycine
(Z,Z)-2,9,16-Heptadecatriene-4,6-diyn-8-ol
11'-Carboxy-gamma-tocotrienol
17beta-Estradiol-3,4-quinone
Lithocholic acid glycine conjugate
Sarcodon scabrosus Depsipeptide
Glycocholic Acid
S-4-benzyl-3-((S)-3-hydroxy-2,2-dimethyloctanoyl)oxazolidin-2-one
PE(18:0/18:3(9Z,12Z,15Z))
DG(16:0/22:6(4Z,7Z,10Z,13Z,16Z,19Z)/0:0)
(±)-Hexanoylcarnitine
(±)-Propionylcarnitine
Isopropyl beta-D-glucoside
Butyryl-L-carnitine
L-Arginine
Physagulin D
Inosine
Hypoxanthine
Seryltyrosine
Spirotaccagenin
1-Hydroxy-10-methylacridone
De-O-methylsterigmatocystin
Riboflavin (Vitamin B2)
N1-(2-Hydroxyethyl)flurazepam
Cis-4,5-Dihydroxy-4,5-dihydropyrene
MOD_1
MOD_2
MOD_3
MOD_4
MOD_5
MOD_6
KBF_M_1
KBF_M_2
KBF_M_3
KBF_M_4
KBF_M_5
KBF_M_6
2
1
0
−1
−2
7
6
5
4
3
2
2
2.5
3
3.5
VIP

(c)

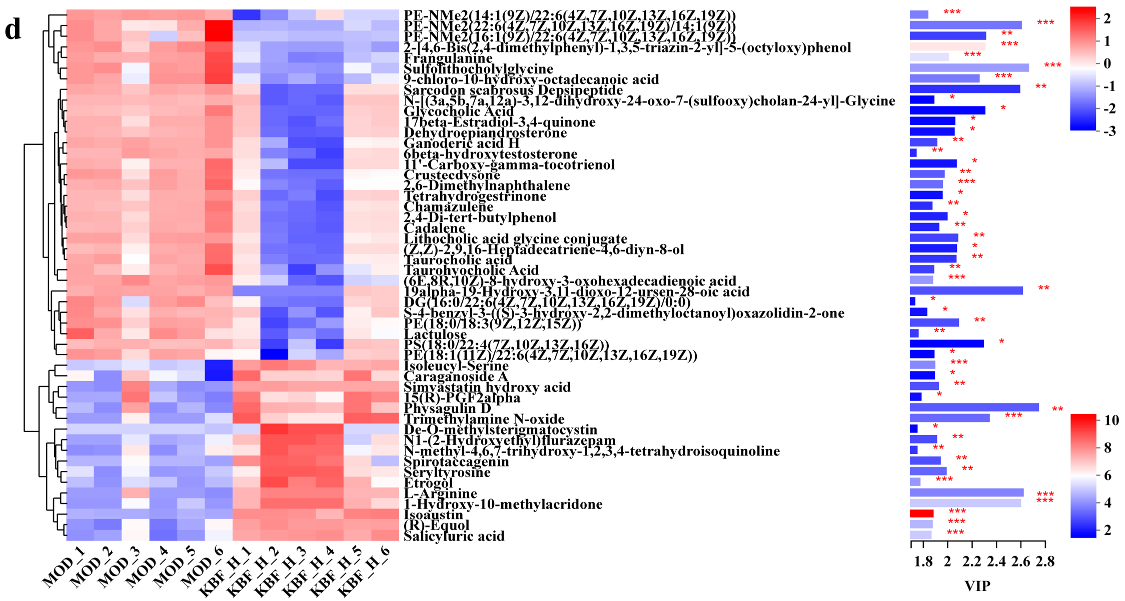
d
PE-NMe2(14:1(9Z)/22:6(4Z,7Z,10Z,13Z,16Z,19Z))
PE-NMe2(22:6(4Z,7Z,10Z,13Z,16Z,19Z)/14:1(9Z))
PE-NMe2(16:1(9Z)/22:6(4Z,7Z,10Z,13Z,16Z,19Z))
2-[4,6-Bis(2,4-dimethylphenyl)-1,3,5-triazin-2-yl]-5-(octyloxy)phenol
Frangulanine
Sulfolithocholylglycine
9-chloro-10-hydroxy-octadecanoic acid
Sarcodon scabrosus Depsipeptide
N-[(3a,5b,7a,12a)-3,12-dihydroxy-24-oxo-7-(sulfooxy)cholan-24-yl]-Glycine
Glycocholic Acid
17beta-Estradiol-3,4-quinone
Dehydroepiandrosterone
Ganoderic acid H
6beta-hydroxytestosterone
11'-Carboxy-gamma-tocotrienol
Crustecdysone
2,6-Dimethylnaphthalene
Tetrahydrogestrinone
Chamazulene
2,4-Di-tert-butylphenol
Cadalene
Lithocholic acid glycine conjugate
(Z,Z)-2,9,16-Heptadecatriene-4,6-diyn-8-ol
Taurocholic acid
Taurohyocholic Acid
(6E,8R,10Z)-8-hydroxy-3-oxohexadecadienoic acid
19alpha-19-Hydroxy-3,11-dioxo-12-ursen-28-oic acid
DG(16:0/22:6(4Z,7Z,10Z,13Z,16Z,19Z)/0:0)
S-4-benzyl-3-((S)-3-hydroxy-2,2-dimethyloctanoyl)oxazolidin-2-one
PE(18:0/18:3(9Z,12Z,15Z))
Lactulose
PS(18:0/22:4(7Z,10Z,13Z,16Z))
PE(18:1(11Z)/22:6(4Z,7Z,10Z,13Z,16Z,19Z))
Isoleucyl-Serine
Caraganoside A
Simvastatin hydroxy acid
15(R)-PGF2alpha
Physagulin D
Trimethylamine N-oxide
De-O-methylsterigmatocystin
N1-(2-Hydroxyethyl)flurazepam
N-methyl-4,6,7-trihydroxy-1,2,3,4-tetrahydroisoquinoline
Spirotaccagenin
Seryltyrosine
Etrogol
L-Arginine
1-Hydroxy-10-methylacridone
Isoaustin
(R)-Equol
Salicyluric acid
MOD_1
MOD_2
MOD_3
MOD_4
MOD_5
MOD_6
KBF_H_1
KBF_H_2
KBF_H_3
KBF_H_4
KBF_H_5
KBF_H_6
1.8
2
2.2
2.4
2.6
2.8
VIP
2
1
0
-1
-2
-3
10
8
6
4
2

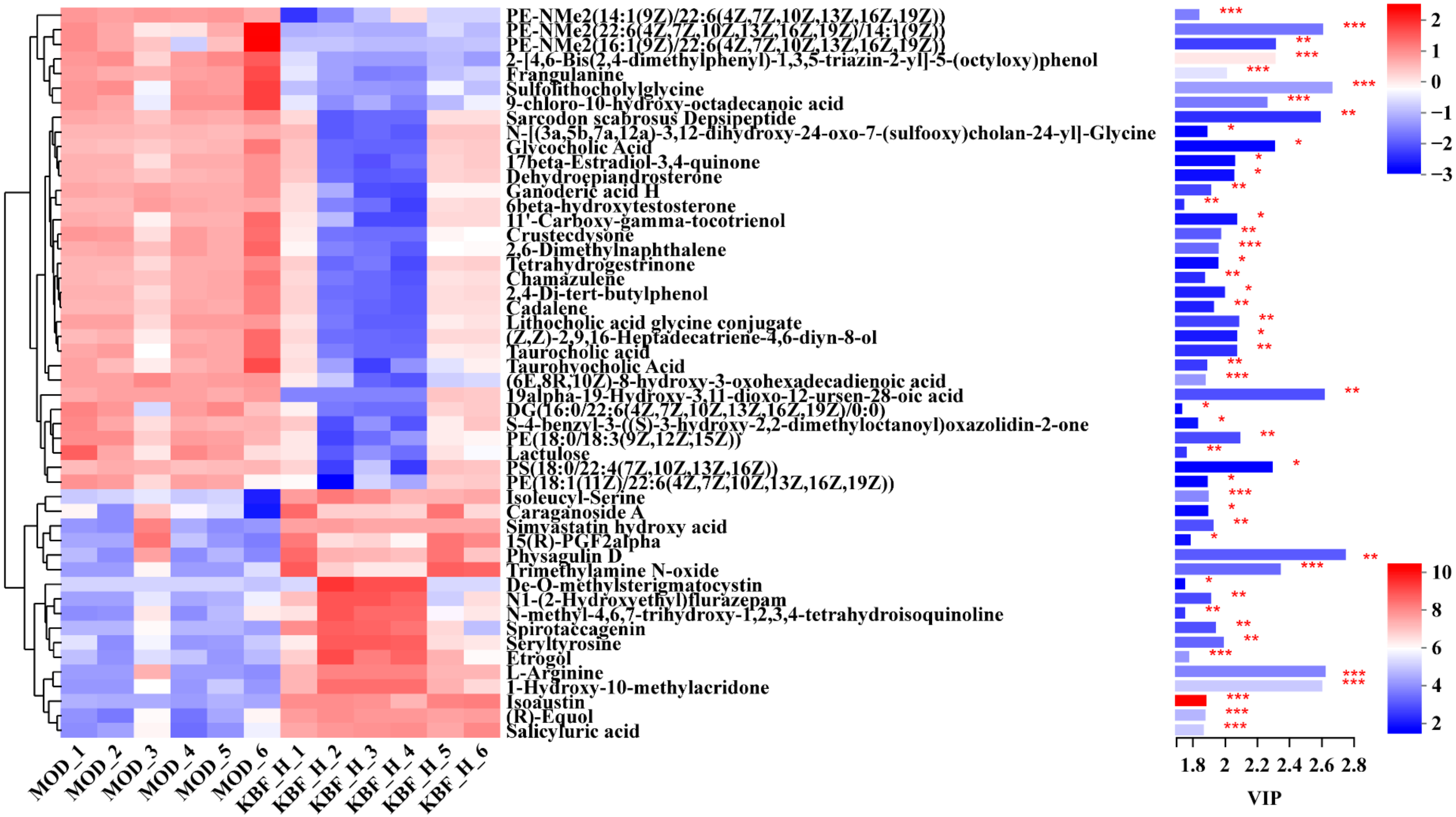

(d)

Figure S1. The heat map of the significant metabolites notably alternated metabolites met the criteria with VIP > 1. (a) CON and MOD; (b) MOD and KBF-L; (c) MOD and KBF-M; (d) MOD and KBF-H.
